# Cardiosphere-Derived Cells from Not Dilated and Dilated Human Myocardium Exhibit Enhanced Metabolic Potential Compared with Conventional Cardiac Mesenchymal Stem/Stromal Cells

**DOI:** 10.3390/ijms27031303

**Published:** 2026-01-28

**Authors:** Daiva Bironaite, Rokas Mikšiūnas

**Affiliations:** Department of Regenerative Medicine, State Research Institute Centre for Innovative Medicine, LT08406 Vilnius, Lithuania

**Keywords:** cardiospheres, mesenchymal stem/stromal cells, cardiac sphere-derived cells, cardiac regeneration, metabolic activity

## Abstract

Dilated cardiomyopathy (DCM) is a major contributor to heart failure and cardiac transplantation. This study investigated the metabolic potential of human myocardium-derived mesenchymal stem/stromal cells (hmMSCs) and subsequently cardiac sphere-derived cells (SDCs) obtained from healthy (non-dilated) and pathological (dilated) myocardial tissues. hmMSCs were isolated using the explant outgrowth method and expanded in monolayer culture. Small round cells loosely attached on hmMSCs were harvested and cultivated as cardiac spheroids for 1–3 days, subsequently obtaining SDCs. The cell morphology, proliferation rate, mitochondrial activity, and intracellular calcium levels were analyzed using flow cytometry, Seahorse metabolic assays, and spectrophotometry, while expression of cell progenitor and cardiac commitment genes were analyzed by quantitative PCR. Both healthy and pathological SDCs demonstrated significantly enhanced mitochondrial function—reflected by increased maximal respiration, ATP production, and coupling efficiency—along with reduced steady-state intracellular calcium levels compared with hmMSCs. SDCs from both healthy and dilated myocardium showed marked upregulation of several cardiac progenitor and lineage-commitment genes relative to hmMSCs. SDCs derived from both healthy and dilated myocardiums possess a more favorable metabolic, progenitor and cardiac commitment profile than conventional hmMSCs. hmMSCs and SDCs from dilated myocardium retain residual metabolic potential, which may be further enhanced under 3D culture conditions.

## 1. Introduction

Heart failure (HF) represents a major global health concern, characterized by increasing prevalence, escalating healthcare costs, and a rising incidence worldwide. Each year, over one million hospitalizations occur in the United States and Europe due to common cardiac diseases [1]. Dilated cardiomyopathy (DCM), a predominant cause of HF, is associated with high morbidity and mortality rates, making it the leading indication for cardiac transplantation [2]. Traditional pharmacological interventions primarily slow disease progression, leaving heart transplantation as the only curative option for advanced HF. Therefore, there is a pressing need to search for novel therapeutic approaches capable of preventing heart failure at the stage of dilated myopathy and improving its regeneration.

The heart was long considered a terminally differentiated organ with minimal regenerative capacity [3]. However, the discovery of cardiac-resident stem-like cells expressing c-KIT and other progenitor markers revealed an intrinsic, though limited, regenerative potential [4,5]. Because c-KIT^+^ and Sca1^+^ populations comprise less than 1% of total cardiac cells, human myocardium-derived MSCs (hmMSCs) have emerged as a promising alternative for cardiac repair promoting regeneration primarily through paracrine signaling, stimulating endogenous repair, modulating immune responses, reducing fibrosis, and/or by limited direct differentiation to cardiomyocytes [4,5]. Although rare transdifferentiation events into cardiomyocytes or vascular cells have been reported [6], clinical translation of hmMSCs remains challenging. This highlights the need for novel strategies—such as genetic, epigenetic, biomaterial-based, or culture-driven approaches—to enhance the regenerative capacity of hmMSCs.

One promising approach to improving the cardiac regenerative potential can be application of cardiospheres and cardiac-sphere-derived cells (SDCs) [7]. These spheroid-derived cells are clonogenic, express stem, endothelial and mesenchymal progenitor cell markers, exhibit self-renewal capacity, and can differentiate into cardiomyocytes and vascular cells both in vitro and in vivo [7]. Over the past decades, cardiac-sphere-derived cells have emerged as one of the most promising allogeneic cell sources for cardiac therapy [8]. SDCs display a heterogeneous gene expression profile, including high levels of stemness-associated genes such as *c-KIT* and *MDR1*, alongside cardiac-specific genes such as *Nkx2.5*, while showing lower expression of mature cardiomyocyte markers [9]. Moreover, SDCs exert cardioprotective, antifibrotic, and immunomodulatory effects [10,11,12,13], but despite evidence of safety following allogeneic SDC injection in post-infarction rat hearts, the risk of immune rejection in humans remains, and cell efficacy may also be undermined by donor age and comorbidities such as myopathies and others [14,15]. Collectively, these findings highlight the importance of SDCs, while underscoring the need for deeper insight into their metabolic biology, especially in healthy versus pathological/dilated human myocardiums.

Efficient cellular metabolism is essential for proper cardiac function, ensuring a continuous supply of oxygen and nutrients. Recent studies have shown that glucose and lactate levels are elevated, while free fatty acid levels decline in hearts with end-stage dilated cardiomyopathy [16]. This finding supports the theory that failing hearts undergo a metabolic shift from oxidative phosphorylation to glycolysis, driven by factors such as impaired mitochondrial dynamics, altered transcriptional regulation of mitochondrial proteins, and other metabolic disturbances [17]. Furthermore, recent metabolomic and transcriptomic advances show that, beyond morphogens and signaling factors, key metabolic pathways—especially oxidative phosphorylation and glycolysis—are crucial for stem cell self-renewal and differentiation [18]. These insights underscore the need to explore novel models for targeted metabolic regulation of human myocardium-derived cells to enhance cardiac regeneration.

Therefore, the aim of this study was to investigate the morphology, proliferation, and metabolic activity, isolated from both healthy and dilated myocardium in comparison to regular hmMSCs. The cardiac commitment genes between human healthy and pathological cardiospheres and SDCs of different passages were also determined. To our knowledge, this is the first study to demonstrate that healthy and dilated myocardium-derived SDCs enhances mitochondrial activity and promotes a more cardiac commitment phenotype, particularly in dilated myocardium-derived SDCs compared with conventional 2D-cultured hmMSCs.

## 2. Results

### 2.1. Isolation and Size Evaluation of Healthy/Not Dilated and Pathological/Dilated Myocardium-Derived hmMSCs and SDCs

**Figure 1:**
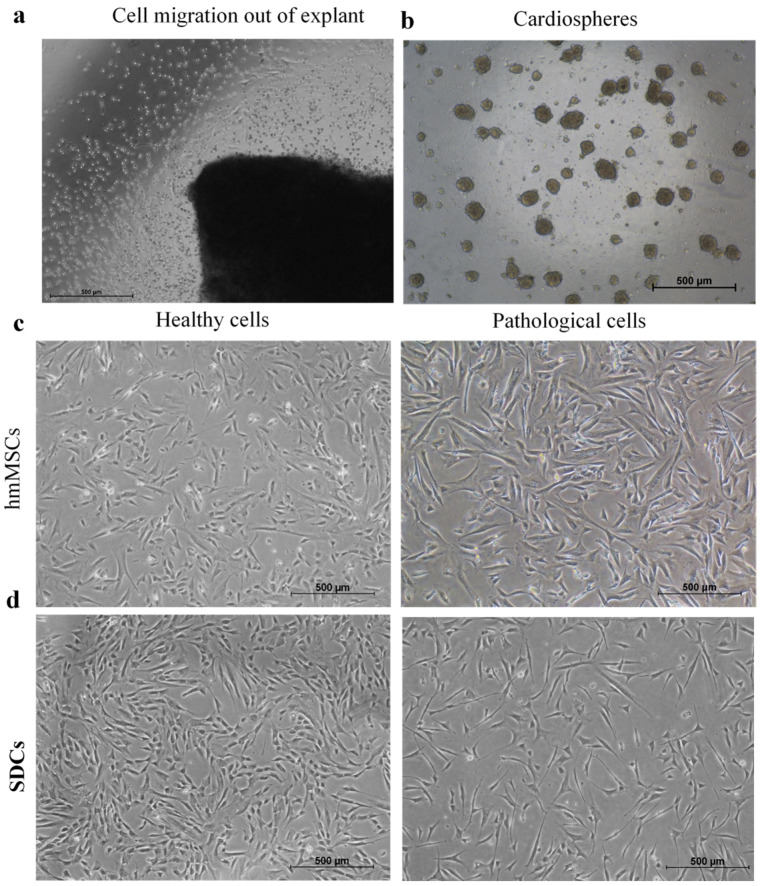
The phenotype of healthy and pathological human myocardium-derived mesenchymal stromal cells (hmMSC) and cardiosphere-derived cells (SDCs). (**a**) hmMSCs migrate out of cardiac explant; (**b**) 3D cardiospheres on non-adherent surface; (**c**) Morphology of healthy and pathological human myocardium-derived hmMSC; (**d**) Morphology of healthy and pathological SDCs. The representative micrograph of healthy hmMSCs was taken from our previous publication [20].

**Figure 2:**
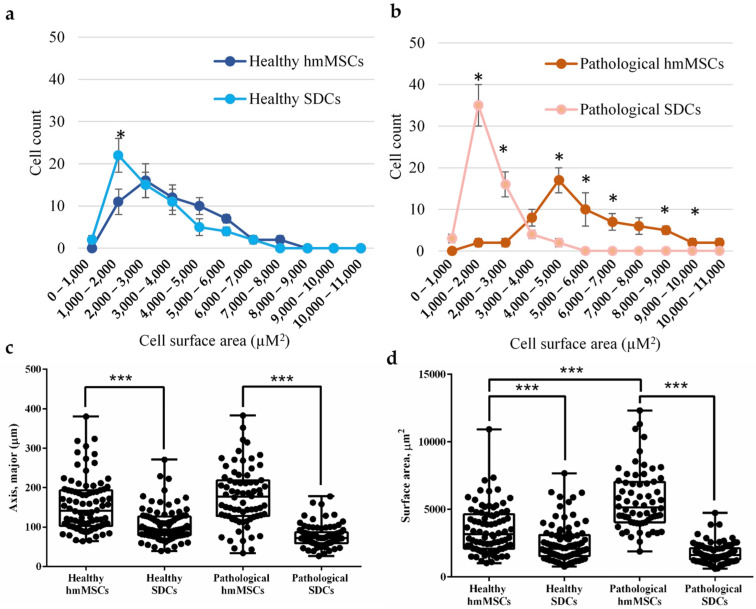
Cell count and surface area of healthy (not dilated) and pathological (dilated) human myocardium-derived mesenchymal stromal cells (hmMSC) and sphere-derived cells. Cell size count of healthy (**a**) and pathological (**b**) hmMSCs; (**c**) Major axis length of healthy and pathological hmMSCs; (**d**) Attachment surface area of healthy and pathological hmMSCs. Data are shown as mean ± standard deviation (SD) and are significant at * *p* ≤ 0.05, *** *p* ≤ 0.001, where n = 50 from three patients for each group measured by ImageJ 1.54p program and calculated using the Graphpad Prism 6 and Excel programs.

The hmMSCs were isolated from healthy and dilated human ventricular myocardium biopsies using the explant outgrowth method. Myocardial biopsies were minced into ~1 mm^3^ fragments, and within 1–3 weeks, hmMSCs migrated out of the tissue and adhered to the fibronectin-coated surface (Figure 1a). The cells were subsequently expanded and used for further experiments (Figure 2c). Our previous studies demonstrated that human heart-derived hmMSCs express typical mesenchymal stem cell surface markers CD90, CD73, and CD105; are negative for CD45, CD10, and CD13; and are capable of differentiating into adipogenic, osteogenic, and chondrogenic lineages [19,20]. In addition, hmMSCs exhibited a tendency toward cardiomyogenic differentiation [19,20].

During explant cultivation, bright, round cells that were loosely attached to the hmMSCs were collected and cultured under three-dimensional (3D) conditions on poly(2-hydroxyethyl methacrylate) (pHEMA)-coated plates using a specialized cardiosphere growth medium (Figure 1b). After three days of cultivation, the resulting cardiospheres were dissociated, and the sphere-derived cells were seeded for further analysis (Figure 1d). Both healthy and pathological SDCs were smaller in size (Figure 1d) compared with their corresponding hmMSCs (Figure 1c,d).

More detailed cell attachment measurements revealed that SDCs from both healthy and pathological samples were approximately twofold smaller in size compared with their corresponding hmMSCs (Figure 2a,b). The cell attachment area also differed between SDCs and hmMSCs in both groups; specifically, the attachment area of healthy SDCs was about twofold smaller than that of healthy hmMSCs, whereas pathological SDCs exhibited an approximately fourfold smaller attachment area compared with pathological hmMSCs (Figure 2c,d). In addition, the attachment area between healthy and pathological hmMSCs differed significantly, as reported previously [20] (Figure 2d). These data suggest that both types of SDCs derived from healthy and DCM myocardium exhibited a reduction in size, which might be associated with a higher cardiac commitment and/or progenitor-like phenotype.

### 2.2. Proliferation of Healthy and Pathological hmMSCs and SDCs

**Figure 3:**
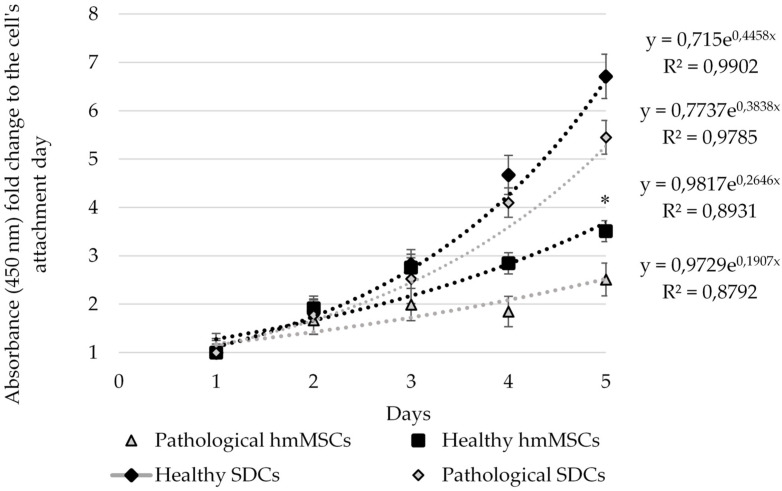
Proliferation rate of healthy and pathological myocardium-derived hmMSC and SDCs. Data are shown as mean ± standard deviation (SD) and are significant at * *p* ≤ 0.05 levels from not less than three repeats (n = 3) of three patients from each group as calculated using the Excel program. Data are significant comparing healthy and pathological hmMSCs and SDCs with corresponding hmMSCs.

The proliferation of healthy and pathological hmMSCs and SDCs was measured using the Cell Counting Kit-8 (CCK-8) assay (Dojindo Molecular Technologies, Inc., Rockville, MD, USA) (Figure 3). In addition, cell proliferation evaluation by direct cell counting showed similar results (Supplementary Materials). Over six days of cultivation, pathological hmMSCs exhibited slightly lower proliferation rates compared with healthy hmMSCs, whereas both healthy and pathological SDCs proliferated significantly faster than their corresponding hmMSCs and showed exponential growth tendency. Overall, the proliferation rate of healthy and pathological SDCs increased approximately twofold compared with the respective hmMSCs.

These data also suggest that healthy hmMSCs and SDCs retained higher initial energetic resources required for proliferation than pathological cells. Both types of SDCs require an adaptation period to 2D growth conditions to achieve an increased proliferative rate.

### 2.3. Mitochondrial Membrane Potential (MMP) and iCa^2+^ of Healthy and Pathological hmMSCs and SDCs

**Figure 4:**
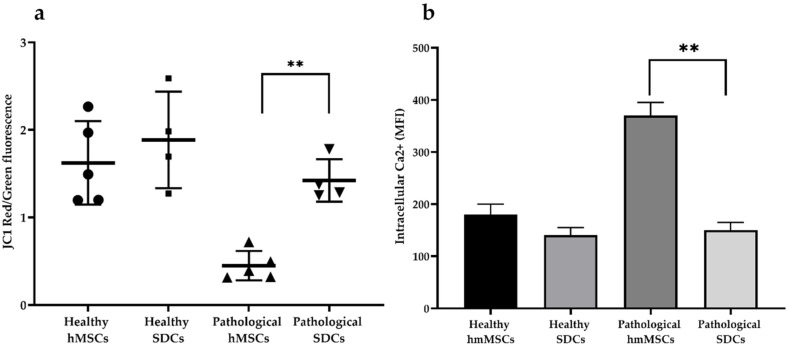
Mitochondrial membrane potential (MMP) and intracellular calcium (iCa^2+^) of healthy and pathological hmMSCs and SDCs. (**a**) Flow cytometric MMP measurement using 5,5′,6,6′-Tetrachloro-1,1′,3,3′-tetraethyl-imidacarbocyanine iodide (JC1) dye; (**b**) Flow cytometric iCa^2+^ measurement using Cal520 (Abcam, Cambridge, UK). Data are shown as mean ± standard deviation (SD). The ** *p* ≤ 0.01, n = 4–5 from five experiments and three patients from each group. Student t test was calculated by Graphpad Prism 6 program.

Mitochondrial membrane potential (MMP) and intracellular calcium (iCa^2+^) levels in healthy and pathological hmMSCs and SDCs were evaluated by staining the cells with JC-1 dye and Cal-520, respectively, followed by flow cytometric analysis (Figure 4). Cells exhibiting elevated MMP showed increased accumulation of JC-1 aggregates (red fluorescence) and a higher red/green fluorescence ratio. In contrast, mitochondria with lower MMP displayed higher green fluorescence from monomeric JC-1 and a lower red/green ratio. Cells with higher intracellular calcium levels demonstrated stronger green fluorescence intensity.

The data showed that pathological hmMSCs had approximately threefold lower mitochondrial activity and a twofold higher steady-state level of iCa^2+^ compared with healthy hmMSCs (Figure 4). Interestingly, pathological SDCs exhibited a significant (~threefold) increase in mitochondrial activity and a reduction in iCa^2+^ levels, approaching those observed in healthy hmMSCs. These findings suggest that SDCs derived from DCM myocardium retain high energetic capacity and exhibit flexible iCa^2+^ signaling, potentially enhanced by 3D cultivation conditions. In contrast, healthy hmMSCs already displayed high mitochondrial activity, which increased only slightly in their corresponding SDCs. Similarly, the iCa^2+^ level in healthy hmMSCs cells was already balanced and did not require significant adjustment in SDCs.

### 2.4. Oxidative Phosphorylation and Glycolysis of Healthy and Pathological hmMSCs and SDCs

**Figure 5:**
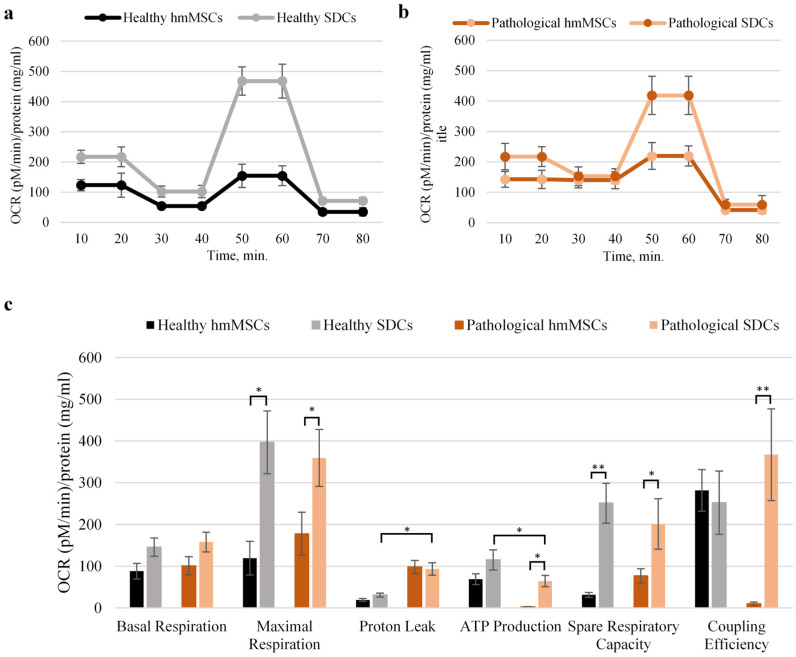
Mitochondrial parameters of healthy and pathological hmMSCs and SDCs. Changes of oxygen consumption rate (OCR) ((pM)/min/mg/mL)) protein in healthy (**a**) and pathological (**b**) hmMSCs and SDCs; (**c**) Calculated mitochondrial parameters in hmMSCs and SDCs of both cell types. Data are shown as mean ± standard deviation (SD). The * *p* ≤ 0.05, ** *p* ≤ 0.01, n = 4 from four experiments and two patients from each group. Student’s t-test was calculated by an Excel program.

The promising impact of cardiospheres on mitochondrial activity prompted a more detailed analysis of oxidative phosphorylation and glycolytic parameters (Figure 5). Therefore, a comprehensive examination of mitochondrial function in both healthy and pathological hmMSCs and SDCs was conducted using the Seahorse XF Analyzer (Agilent Technologies, North Billerica, MA, USA). This system enables the assessment of mitochondrial respiration and glycolytic activity by measuring the oxygen consumption rate (OCR) and extracellular acidification rate (ECAR), respectively.

The Seahorse data confirmed previous observations: pathological hmMSCs exhibited significantly lower coupling efficiency (9.1 vs. 281.22 pM/min/mg protein) and ATP production (19.2 vs. 98.1 pM/min/mg protein), as well as a higher proton leak (98.14 vs. 19.24 pM/min/mg protein) compared with healthy hmMSCs (Figure 5). Interestingly, maximal respiration (177.68 vs. 119.39 pM/min/mg protein) and spare respiratory capacity (76.79 vs. 31.23 pM/min/mg protein) were higher in pathological hmMSCs than in healthy cells, suggesting that pathological cells retain mitochondrial energetic reserves that can potentially be activated for regenerative purposes.

Cultivation of both healthy and pathological cardiac progenitors in 3D cardiospheres, followed by assessment of their derived SDCs, revealed a significant increase in ATP production in both SDC types (from 68.91 to 115.08 pM/min/mg protein in healthy, and from 3.4 to 64.4 pM/min/mg protein in pathological cells). Coupling efficiency was also markedly upregulated only in pathological SDCs (from 9.1 to 366.97 pM/min/mg protein), while proton leak remained nearly unchanged between the two SDC types. Additionally, 3D cardiosphere cultivation enhanced basal respiration, maximal respiration, and spare respiratory capacity in both healthy and pathological SDCs.

**Figure 6:**
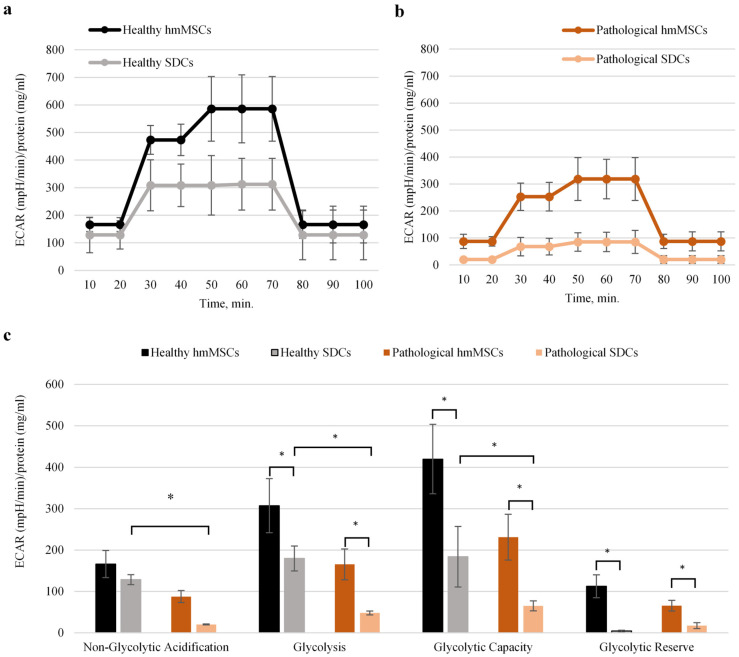
Glycolytic activity of healthy and pathological hmMSCs and SDCs. Changes in extracellular acidification rate (ECAR) ((mpH)/min/mg/mL)) of protein in healthy (**a**) and pathological (**b**) hmMSCs and SDCs; (**c**) Calculated glycolytic parameters in hmMSCs and SDCs. Data are shown as mean ± standard deviation (SD). Data are shown as mean ± standard deviation (SD). The * *p* ≤ 0.05, n = 4 from four experiments and two patients from each group. Student’s t-test was calculated by an Excel program.

These findings suggest that 3D cardiosphere cultivation enhances nearly all mitochondrial bioenergetic parameters, except for the proton leak, in SDCs compared with hmMSCs. Mitochondrial proton leak refers to protons re-entering the mitochondrial matrix independently of ATP synthase, which might be related to the intactness of mitochondrial inner membrane; this phenomenon may result from alterations in mitochondrial inner membrane lipid composition and/or structural disruptions. In such cases, proton leak reflects pathological uncoupling, not physiological regulation. The impact of 3D cardiosphere culture on glycolysis in healthy and pathological SDCs was also investigated (Figure 6a,b). The results confirmed that 2D-cultured pathological hmMSCs exhibited lower glycolytic activity compared with 2D-cultured healthy hmMSCs (Figure 6). Furthermore, 3D cardiosphere did not significantly improve glycolytic stress parameters in either healthy or pathological SDCs compared with their corresponding hmMSCs. These findings indicate that the reduced glycolytic activity observed in pathological hmMSCs and SDCs is associated with their slower proliferation rate compared with healthy cells (Figure 2). The improved mitochondrial activity accompanied by decreased glycolytic activity suggests a metabolic shift of SDCs from proliferation toward differentiation and regeneration.

### 2.5. Cardiac Commitment Gene Expression Profile of Healthy and Pathological Cardiospheres and SDCs Versus hmMSCs

**Figure 7:**
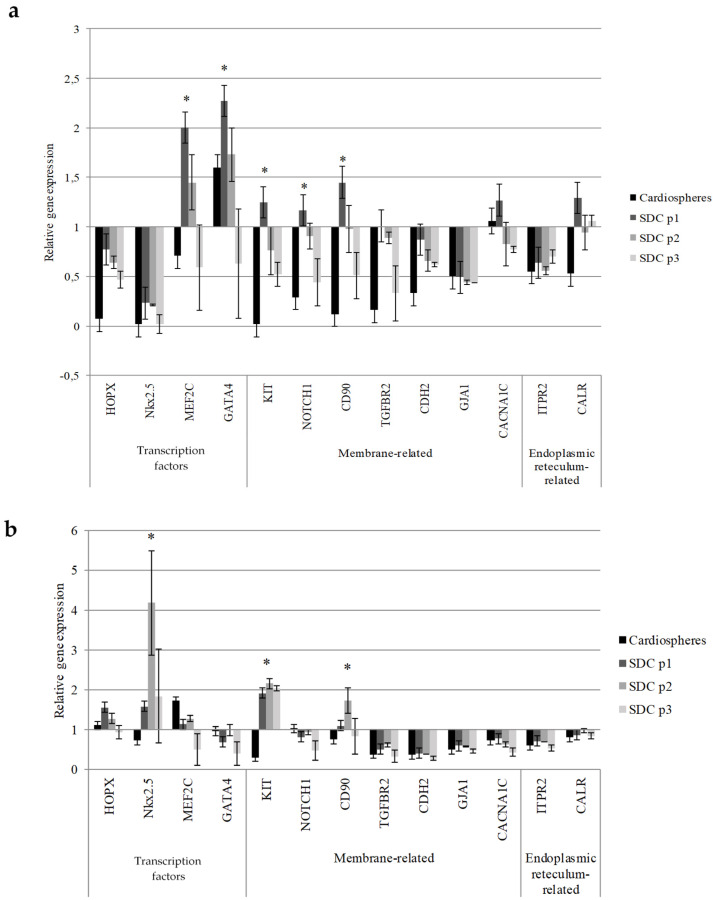
Expression of cardiac commitment genes in healthy (not dilated) and pathological (dilated), cardiospheres and sphere-derived cells (SDCs) vs. hmMSCs. (**a**) Relative gene expression in healthy SDCs and cardiospheres vs. hmMSCs; (**b**) Relative gene expression in pathological/dilated SDCs and cardiospheres vs. hmMSCs. The expression of genes is shown as (2^−ΔCT^ × 100,000,000). Data are shown as mean ± standard deviation (SD). The * *p* ≤ 0.05, n = 3 from three experiments and two patients from each group. Student’s t-test was calculated by Excel program.

The observed differences in cell morphology, proliferation, mitochondrial membrane potential, and metabolic activity between hmMSCs and SDCs prompted an investigation of the expression of cardiac commitment-related transcription factors (*HOPX*, *Nkx2.5*, *MEF2C*, *GATA4*), cell membrane (*c-KIT*, *NOTCH1*, *CD90*, *TGFBR2)*, *CDH2*, *GJA1*, *CACNA1C*) and endoplasmic reticulum-associated genes (*ITPR2*, *CALR*) in cardiospheres and SDCs vs. hmMSCs (Figure 7).

Gene expression analysis revealed that healthy SDCs exhibited significantly higher expression of stemness-related genes such as *c-KIT*, *NOTCH1*, and *THY1*, whereas pathological SDCs showed increased expression of *c-KIT* and *THY1* only (Figure 7). These findings suggest that SDCs from both healthy and pathological myocardiums retain and enhance their mesenchymal/stem-like phenotype, characteristic of progenitor cells. The 3D cardiosphere culture likely promotes a more primitive and regenerative phenotype, maintaining high expression of *THY1* along with other stemness-associated markers (*c-KIT*, *HOPX*, and *NOTCH1*) (Figure 7).

Furthermore, the expression of cardiac commitment markers such as *MEF2C* and *GATA4* was significantly upregulated in healthy SDCs, whereas *NKX2-5* was the only commitment factor significantly increased in dilated myocardium-derived SDCs (Figure 7). In addition, the expression of intracellular calcium regulators such as *CACNA1C* (Cav1.2) and *CALR* was also higher in healthy SDCs than in pathological counterparts (Figure 7).

It is noteworthy that gene expression upregulation occurred earlier (at passage 1) in healthy SDCs, while in pathological SDCs, similar changes appeared later (at passage 2). These findings indicate that healthy myocardium-derived SDCs likely possess a more stable differentiation and regeneration program, supported by superior metabolic and mitochondrial functions. Nevertheless, SDCs derived from dilated myocardium still retain residual regenerative potential, which could potentially be enhanced through targeted modulation.

## 3. Discussion

In recent decades, several clinical trials have investigated cell therapy using intracoronary infusion of autologous SDCs in post-MI patients [14], allogeneic SDCs in patients with progressive heart failure [21] or intracoronary administration of allogeneic SDCs in patients with post-MI left ventricular dysfunction [22]. Although SDC treatment was safe, it did not significantly reduce scar size compared with placebo at 6 months. However, reductions in left ventricular volumes and NT-proBNP levels indicated some disease-modifying bioactivity of SDCs. Despite the apparent therapeutic benefits of SDCs in cardiac regeneration, several questions remain regarding their biology—particularly when comparing cells derived from healthy versus dilated myocardium. One key question is whether it is possible to enhance the energetic and cardiac commitment status of autologous human dilated myocardium-derived SDCs compared with hmMSCs, and if so, which parameters change the most and how.

Human myocardium-derived stem/stromal cells (hmMSCs) can be isolated from biopsies of both healthy and dilated ventricular myocardium. In our previous studies, we demonstrated that hmMSCs express typical mesenchymal stem cell surface markers and are capable of differentiating into osteogenic, adipogenic, chondrogenic, and cardiomyogenic lineages [19,20]. Additionally, we investigated the epigenetic acetylation levels of healthy and dilated hmMSCs and showed that histone deacetylase inhibitors can enhance their regenerative potential [19,20]. In the present study, we compared healthy and dilated myocardium-derived hmMSCs, cardiospheres, and cardiosphere-derived cells (SDCs) to assess their morphological characteristics, proliferation, energetic/metabolic profiles, and cardiac commitment-related gene reprogramming.

Other studies have shown that three-dimensional (3D) cell cultures provide a more physiologically relevant model than traditional two-dimensional (2D) systems, enabling natural cell-environment interactions and more accurate predictions of biological behavior and drug responses [23,24,25]. It has also been reported that the transition from 2D to 3D culture is accompanied by metabolic reprogramming, characterized by increased mitochondrial respiration and glycolytic activity [26]. This study demonstrates that in pathological SDCs, the decreased mitochondrial membrane potential (MMP) and elevated intracellular Ca^2+^ levels in pathological hmMSCs were restored nearly to those of healthy hmMSCs. SDCs also exhibited higher oxidative phosphorylation and lower glycolytic activity compared with hmMSCs of both types. Detailed metabolic analysis revealed that while pathological hmMSCs showed reduced mitochondrial coupling and ATP production—both improved in SDCs—the increased proton leak observed in pathological hmMSCs persisted in SDCs. Interestingly, pathological hmMSCs exhibited higher spare respiratory capacity and maximal respiration than healthy hmMSCs, suggesting a retained energetic potential in DCM-derived hmMSCs. The enhancement of oxidative phosphorylation (OXPHOS) as an alternative energy production pathway to glycolysis in 3D cultures has been proposed to reflect increased energy demands and necessary metabolic adaptation to the 3D microenvironment [27,28].

Morphological and proliferation analyses revealed new tendencies related to the glycolytic activity of hmMSCs and SDCs. In this study, both healthy and pathological SDCs were smaller in size and proliferated faster than hmMSCs, while exhibiting significantly lower glycolytic activity. According to the generally accepted theory, adult stem cells display metabolic flexibility regulated by their adhesion status—adherent cells primarily utilize glycolysis, whereas suspended cells depend on oxidative phosphorylation (OXPHOS) to meet their ATP demands [29]. Our data suggest that SDCs rely more heavily on mitochondrial OXPHOS than on glycolysis to support their energy requirements. Enhanced mitochondrial function, biogenesis, or metabolic efficiency in SDCs may enable greater ATP production per glucose molecule, thereby sustaining higher proliferation despite reduced glycolytic flux. It is possible that the metabolic transition from OXPHOS to glycolysis in SDCs requires longer incubation under 2D culture conditions. Alternatively, the observed lower glycolytic rate may reflect a more mature and energetically balanced phenotype, consistent with the partial cardiac commitment of SDCs compared with undifferentiated hmMSCs [30].

Metabolic changes in SDCs are often accompanied by modulation of transcription factors and other genes related to cardiac progenitor identity and lineage commitment. In this study, the most pronounced expression of stemness-related genes—*c-KIT*, *NOTCH1*, and *THY1* (CD90)—was observed in healthy SDCs at passage one, whereas in pathological SDCs, increased expression of *c-KIT* and *THY1* was detected only at passage two. These data indicate that healthy SDCs more readily shift toward a primitive, progenitor-like state than pathological SDCs. Activation of the c-KIT receptor has been shown to promote cell survival and proliferation in both human and murine stem cells and cardiomyocytes [31]. Moreover, c-KIT^+^ cardiac progenitors are recognized as primitive stem cells with multilineage differentiation potential [32]. Clinically, the Phase I SCIPIO trial demonstrated the safety of autologous c-KIT^+^ cardiac cell administration in patients with ischemic heart failure [33]. However, other studies reported that endogenous c-KIT^+^ cells generate cardiomyocytes at a functionally insignificant level [34] and that transplanted c-KIT^+^ cells in infarcted hearts do not significantly differentiate into mature cardiomyocytes [33]. These findings suggest that activation of c-KIT alone may be insufficient to induce effective cardiac regeneration and likely requires the cooperative activation of other stemness-related genes such as NOTCH1, CD90, or additional regulatory factors.

For many years, Thy-1/CD90 was used primarily as a marker to define mesenchymal stem cells (MSCs). However, recent studies have shown that Thy-1 also possesses signaling functions and plays a critical role in determining MSC fate by promoting osteogenic differentiation, while inhibiting adipogenic differentiation [35]. It has also been reported that THY1 expression in fibrotic cardiac tissue is not associated with fibroblast differentiation, but may serve as a marker of fibroblast proliferation [36]. In addition, activation of the Notch signaling pathway has been observed in CD90^+^ liver cancer cells, where it promotes cell invasion, migration, and the expression of stemness-related genes [37,38]. However, the potential cooperative role of the Notch pathway with other stemness-associated genes such as c-KIT and CD90 in cardiac regeneration remains to be elucidated.

Finally, the expression of cardiomyogenic commitment genes *MEF2C* and *GATA4* was significantly upregulated in healthy SDCs, whereas in pathological SDCs, only the early cardiomyogenic differentiation gene *NKX2.5* was elevated. The transcription factors *MEF2C* and *GATA4* are key regulators of cardiomyocyte maturation and structural gene expression. A recent study demonstrated that transfection of *GATA4* and *MEF2C*, individually or in combination, induced cardiac differentiation in human umbilical cord-derived MSCs, leading to increased expression of cardiac genes such as *NKX2.5*, myosin heavy chain (*MYH6*, *MYH7*), connexin-43 (*GJA1*), and cardiac proteins including GATA4, NKX2.5, cardiac troponin T, and connexin-43 [39]. In this study, the higher expression of *MEF2C* and *GATA4* in healthy SDCs suggests that these cells are further along the cardiomyogenic differentiation pathway, reflecting a more mature or committed cardiac phenotype. In contrast, *NKX2.5*—an early cardiac progenitor marker and one of the first transcription factors expressed during cardiac lineage specification [40]—was significantly upregulated in pathological SDCs, indicating that these cells may be arrested at an earlier developmental stage compared with healthy SDCs, maintaining a more primitive or progenitor-like phenotype rather than progressing toward full cardiomyogenic differentiation. DCM-derived SDCs may therefore require stronger stimuli than 3D cultivation alone to enhance cardiac commitment. The chronic stress, fibrosis, and/or metabolic impairments in dilated myocardium may further delay or inhibit normal differentiation, maintaining cells in a compensatory, progenitor-activated state. This area warrants further detailed investigation.

## 4. Materials and Methods

### 4.1. Isolation of hmMSC and Growing in 2D Conditions

Human healthy ventricular biopsies were obtained from 65–75-year-old men patients without mitral valve disease, non-dilated left ventricle, and preserved its function. Human dilated myocardium biopsies were obtained from 65–75-year-old men with mitral valve disorders and dilated left ventricles showing reduced ejection fraction (<45–50%). Healthy biopsies were obtained from inside the heart using a thin catheter; meanwhile, dilated myocardium biopsies were obtained from leftover material after mitral valve change operation. Cell isolation was performed according to previously described protocols with minor modifications [20]. Briefly, myocardial specimens were minced into fragments smaller than 1 mm^3^, thoroughly washed with phosphate-buffered saline (PBS) containing 2% antibiotics, and partially digested with trypsin. The tissue fragments were then cultured as explants on fibronectin-coated (2 mg/mL) six-well plates in Iscove’s Modified Dulbecco’s Medium (IMDM) supplemented with 20% fetal bovine serum (FBS), 100 U/mL penicillin G, and 100 U/mL streptomycin (Thermo Fisher Scientific, Waltham, MA, USA). Within approximately 1–3 weeks, a layer of stromal-like cells emerged from the explants. Once near confluence, cardiac outgrowth cells were detached using trypsin and transferred to 75 cm^2^ flasks coated with 0.2% gelatin for further expansion. The remaining tissue fragments were re-plated onto fibronectin-coated dishes to allow continued outgrowth formation. Cardiac outgrowth cells could be harvested up to four times from the same explants. Cell counting for the experiments was performed with Fast-Read 102^®^ plastic chamber (Biosigma, Cona, Italy).

### 4.2. Formations of Cardiospheres

During explant outgrowth phase, small round loosely attached cells were harvested with 0.05% of trypsin and seeded on the poly-HEMA poly(2-hydroxyethyl methacrylate)-coated 96 well plates for the formation of cardiospheres. Collected cells were grown in special cardiosphere medium for 3 days 35% IMDM/65% DMEM-Ham’s F-12 (GIBCO), 3.5% Embryonic stem cell FBS, 2% B27, 100 U/mL penicillin G, 100 U/mL streptomycin (Thermo Fisher Scientific, Waltham, MA, USA), 0.1 mmol/L 2-mercaptoethanol, 10 ng/mL EGF and 20 ng/mL bFGF (Sigma Aldrich, St. Louis, MO, USA), 10 ng/mL Cardiotrophin-1 (Biochrom, Cambridge, UK), 1 unit/mL thrombin (Merck KGaA, Damstadt, Germany), to form 3D cardiospheres [29]. After this, cardiospheres were dissociated with 0.25% of trypsin-EDTA to obtain cardiosphere-derived cells (SDCs) that were seeded on the 25 cm^2^ flask coated with the 0.2% gelatine for the further growth in IMDM supplemented with 10% FBS and 100 U/mL penicillin G, 100 U/mL streptomycin. In this study 1st to 3rd passage of SDCs were used for the further experiments.

### 4.3. Proliferation Measurements

A total of 5000 cells were calculated with Fast-Read^®^ 102 (Biosigma, Cona, Italy) and seeded in 24-well plate. The proliferation of ventricle-derived hmMSCs and SDCs was monitored continuously over a period of six days using the CCK-8 kit, as recommended by the manufacturer (Dojindo, Kumamoto, Japan). Briefly, 12.5 µL of CCK-8 solution was introduced to 500 µL of fresh growth medium, followed by a 3 h incubation at 37 °C followed by absorbance measurement at 450 nm using the SpectraMax i3 spectrophotometer (Molecular Devices, San Jose, CA, USA).

### 4.4. Evaluation of Intracellular Calcium Measurement Using Flow Cytometry

For flow cytometry analysis, cells were seeded into six-well plates at a density of 50,000 cells per well. After reaching the desired confluence, cells were detached using trypsin, transferred to flow cytometry tubes, and centrifuged at 600× *g* for 5 min. The cell pellets were stained with the 3 μM of calcium-specific fluorescent dye Cal-520 (Santa Cruz Biotechnology, Dallas, TX, USA) in IMDM with 1% PEST and incubated at 37 °C with 5% CO_2_ for 30 min. Following incubation, cells were washed twice with 1% BSA PBS solution and centrifuged at 600× *g* for 5 min. The supernatant was discarded, and cells were resuspended in 300 μL of PBS containing 1% BSA. Each sample was analyzed in triplicate, and fluorescence intensity was measured using a FACSAria III flow cytometer (BD Biosciences, San Jose, CA, USA).

### 4.5. Evaluation of Mitochondria Potential Using Flow Cytometry

The confluent cells monolayer was harvested with trypsin; cells were stained with 2 µM of JC1 in IMDM with 1% PEST at 37 °C with 5% CO_2_ for 30 min. After staining, cells were washed twice and resuspended in 300 μL of 1% BSA PBS solution. Flow cytometry was performed with BD FACSAria II instrument (BD Biosciences, San Jose, CA, USA); data were analyzed with BD FACSDiva software 6.1 (BD Biosciences, San Jose, CA, USA).

### 4.6. Metabolic Measurements with Seahorse

Seahorse XFp Analyzer (Agilent Technologies, Santa Clara, CA, USA) was used to evaluate the oxygen consumption rate (OCR) and extracellular acidification rate (ECAR) of hmMSCs and SDCs. The cells were seeded at a density of 20,000 cells per well on Seahorse XFp culture plates a day before the measurements. Seahorse medium (Agilent Technologies, Santa Clara, CA, USA) was prepared following the manufacturer’s recommendations. For OCR measurements, the medium contained final concentrations of 10 mM glucose, 2 mM glutamax, and 1 mM sodium pyruvate. Mitochondrial parameters were assessed by sequentially introducing 10 µM oligomycin, 20 µM carbonyl cyanide 4-(trifluoromethoxy)phenylhydrazone (FCCP), and 5 µM of rotenone (Agilent Technologies, Santa Clara, CA, USA).

In ECAR measurements, 1 mM of glutamine was added to the Seahorse medium. Glycolytic stress was evaluated by sequentially adding 100 mM glucose, 50 µM oligomycin, and 500 mM 2-deoxyglucose (DG) (Agilent Technologies, Santa Clara, CA, USA). First passage hmMSCs and SDCs were used for this experiment. After the experiment, all samples were lysed in lysis buffer (50 mM Tris HCl, pH = 6.8, 10% glycerol, 1% SDS), followed by centrifugation at 20,000× *g* and + 4 °C for 15 min. The protein concentration was determined using the Pierce™ Modified Lowry Protein Assay Kit (Thermo Fisher Scientific, Waltham, MA, USA). Energetic parameters were normalized by protein concentration and expressed as pM/min/mg/mL protein for OCR measurements and mpH/min/mg/mL protein for ECAR measurements.

### 4.7. Real-Time RT-PCR

RNA was purified approximately from 2 × 10^5^ hmMSCs, SDCs or cardiospheres using GeneJetTM RNA purification kit (Thermo Fisher Scientific, Waltham, MA, USA). A total of 500 ng of RNA was incubated with DnaseI at 37 °C for 30 min, then reaction was stopped by adding 50 mM EDTA and heating at 65 °C for 5 min. (Thermo Fisher Scientific, Waltham, MA, USA). Reverse transcription was performed with a High Capacity cDNA reverse transcription kit (Thermo Fisher Scientific, Waltham, MA, USA). Real-time PCR was performed in triplicate using the 2× Maxima Probe qPCR Master Mix (Thermo Fisher Scientific, Waltham, MA, USA) on a AriaMx Real-Time PCR Machine (Agilent Technologies, Santa Clara, CA, USA) with an annealing temperature of 60 °C. 

Expression of transcription factors (*HOPX*, *GATA4*, *NKX2-5*, *MEF2C*), cell membrane (*cKIT*, *NOTCH1*, *THY1*, *TGFBRII*, *CDH2*, *GJA1*, *CACNA1C*), and endoplasmic reticulum-related (*ITPR2* and *CALR*) genes was determined (Thermo Fisher Scientific, Waltham, MA, USA) (Table 1). *HPR1*, *ACTB*, *GAPDH*, and *B2M* housekeeping genes were evaluated for expression stability across different cell types, patients, and culturing conditions using equal amounts of RNA for cDNA synthesis. Among these genes, B2M showed lowest coefficient of variation and NormFinder stability value; therefore, it was selected for gene expression normalization.

### 4.8. Statistical Analysis

The statistical analysis was performed using the Excel software (Microsoft Corporation, Redmont, WA, USA) and Graphpad Prism 6.01 (GraphPad Software, San Diego, CA, USA). The data are expressed as means ± standard deviation (mean ± SD) based on not less than three replicates from two to three healthy and dilated human myocardium-derived hmMSCs and SDCs. Significance of the data was determined using Student’s t-test, and significance levels were indicated at * *p* ≤ 0.05 or ** *p* ≤ 0.01 levels.

## 5. Conclusions

Our findings demonstrate that SDCs derived from both healthy and dilated atrial myocardium possess a more favorable metabolic, progenitor and cardiac commitment profile than conventional hmMSCs.

Although some mitochondrial features—such as proton leak—remained suboptimal, the overall enhancement in ATP production, coupling efficiency, and respiratory capacity underscores the metabolic advantages of both types of SDCs over conventional hmMSCs. The higher metabolic activity of cardiac SDCs over hmMSCs may also be related to the stronger expression of some stemness- and cardiomyogenesis-associated genes.

Collectively, while DCM-derived hmMSCs exhibited signs of impaired mitochondrial function, lower proliferation and altered calcium homeostasis, the SDC phenotype partially restored several key bioenergetic parameters, highlighting the residual metabolic potential of pathological cells, which can be targeted stimulated. Healthy SDCs possess a more mature or committed cardiac phenotype, whereas dilated myocardium-derived SDCs may require supplementary external stimuli, in conjunction with or beyond cardiosphere culture, to further achieve enhanced cardiac commitment.

A limitation of this study relates to the intrinsic differences in cellular homogeneity between SDCs and hmMSCs and the potential impact this could have on data interpretation. hmMSCs are known to represent a heterogeneous population with some variability in cell surface marker expression, differentiation state, and/or metabolic activity [54], whereas SDCs appear more phenotypically uniform under the conditions used here. The widely used definition of typical MSC surface markers may not be universally applicable, as markers expression is highly dependent on the tissue of origin [55,56].

This disparity in population homogeneity may partly account for the observed differences in both metabolomic and transcriptomic profiles, as bulk analyses inevitably reflect averaged signals that can be disproportionately influenced by dominant subpopulations. However, SDCs displayed highly proliferative, secretory and immunomodulatory properties that can also be found in activated or inflammatory cell types [57]. Moreover, sc-RNAseq data from human right atrial biopsies in comparison with SDCs uncovered transcriptomic similarities between SDCs and cardiac fibroblasts, also named as hmMSCs, but not with cardiac progenitor cells derived from human-induced pluripotent stem cells [57]. While our study provides valuable comparative insights, future work incorporating single-cell-level analyses in more refined phenotypic and functional stratification will be necessary to disentangle heterogeneity-driven cardiac regenerative effects from genuine biological differences between SDCs and hmMSCs.

## Figures and Tables

**Table 1:** Analyzed gene function in heart development and disease.

Protein (Gene)	Function Summary	Taqman Assay Id	Key Reference (DOI)
	Transcription Factors		
*HOPX*	Regulates cardiomyocyte gene networks via protein–protein interactions rather than direct DNA binding.	Hs04188695_m1	[41]
*NKX2-5*	Essential for early heart development and cardiac conduction system formation.	Hs00231763_m1	[42]
*MEF2C*	Controls cardiomyocyte differentiation and heart morphogenesis, including outflow tract formation.	Hs00231149_m1	[43]
*GATA4*	Zinc-finger transcription factor required for cardiac morphogenesis and cardiomyocyte survival.	Hs00171403_m1	[44]
	Cell Membrane-Related Proteins		
*c-KIT* (CD117)	Tyrosine kinase receptor involved in stem/progenitor cell maintenance and cardiac repair.	Hs00174029_m1	[45]
NOTCH1	Regulates heart development and cardiomyocyte/vascular differentiation.	Hs01062014_m1	[46]
*THY1* (CD90)	Cell surface glycoprotein involved in cardiac fibroblast regulation and cell adhesion.	Hs00174816_m1	[47]
*TGFBR2*	TGF-β receptor critical for heart vessels and descending thoracic aorta development	Hs00234253_m1	[48]
*CDH2* (N-cadherin)	Essential for cardiomyocyte adhesion and intercalated disk integrity.	Hs00983056_m1	[49]
*GJA1* (Connexin43)	Main gap junction protein for cardiomyocyte electrical coupling.	Hs00748445_s1	[50]
CACNA1C (*CaV1.2*)	L-type Ca^2+^ channel subunit essential for cardiac excitation–contraction coupling.	Hs00167681_m1	[51]
	Endoplasmic Reticulum-Related Proteins		
*ITPR2*	ER IP3 receptor regulating Ca^2+^ release in cardiac signaling and hypertrophy.	Hs00181916_m1	[52]
*CALR*	ER chaperone regulating calcium homeostasis and cardiac development.	Hs00189032_m1	[53]

## Data Availability

The original contributions presented in this study are included in the article. Further inquiries can be directed to the corresponding author.

## Supplementary Materials

The following supporting information can be downloaded at: https://www.mdpi.com/article/10.3390/ijms27031303/s1.

## Abbreviations

The following abbreviations are used in this manuscript:

SDCs	Sphere-derived cells
hmMSCs	Human cardiac myocardium-derived mesenchymal stem/stromal cells
DCM	Dilated cardiomyopathy
ATP	Adenosine triphosphate
HF	Heart failure
OCR	Oxygen consumption rate
ECAR	Extracellular acidification rate
MMP	Mitochondrial membrane potential
KIT	KIT Proto-Oncogene, Receptor Tyrosine Kinase
NKX2-5	NK2 Homeobox 5
MDR1	ATP Binding Cassette Subfamily B Member 1
HOPX	HOP Homeobox
NOTCH1	Notch Receptor 1
TGFBR2	Transforming Growth Factor Beta Receptor 2
CDH2	Cadherin 2
GJA1	Gap Junction Protein Alpha 1
CACNA1C	Calcium Voltage-Gated Channel Subunit Alpha1 C
ITPR2	Inositol 1,4,5-Trisphosphate Receptor Type 2
CALR	Calreticulin
