# Supplementary figures and images for: Cardiosphere-Derived Cells from Not Dilated and Dilated Human Myocardium Exhibit Enhanced Metabolic Potential Compared with Conventional Cardiac Mesenchymal Stem/Stromal Cells
Source: Int J Mol Sci. 2026 Jan 28;27(3):1303. doi: 10.3390/ijms27031303 (PMC12898092; doi:10.3390/ijms27031303)

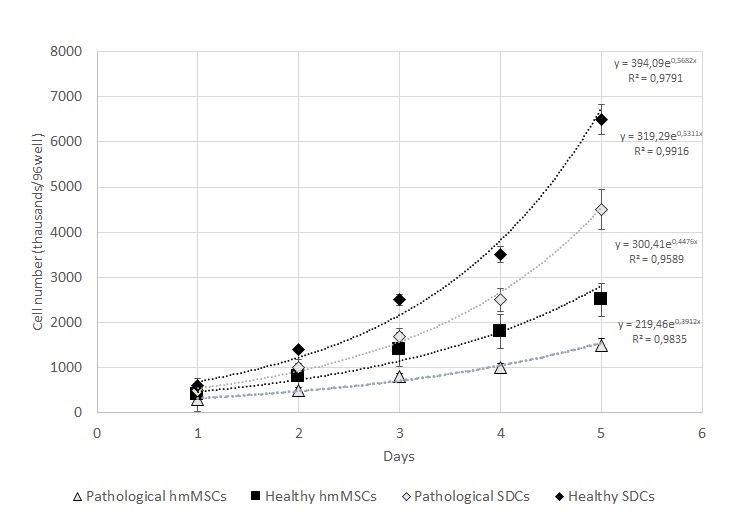

Supplement: Supplementary file 1 [file ijms-27-01303-s001.zip › ijms-4017119-supplementary.jpg]
